# Retraction: Synthesis of deuterated isopentyl pyrophosphates for chemo-enzymatic labelling methods: GC-EI-MS based 1,2-hydride shift in epicedrol biosynthesis

**DOI:** 10.1039/d0ra90009e

**Published:** 2020-01-27

**Authors:** Madhukar S. Said, Govinda R. Navale, Jayant M. Gajbhiye, Sandip S. Shinde

**Affiliations:** Organic Chemistry Division, CSIR-National Chemical Laboratory (CSIR-NCL) Dr Homi Bhabha Road Pune-411008 India ss.shinde@ncl.res.in; Academy of Scientific and Innovative Research (AcSIR) Ghaziabad 201 001 India

## Abstract

Retraction of ‘Synthesis of deuterated isopentyl pyrophosphates for chemo-enzymatic labelling methods: GC-EI-MS based 1,2-hydride shift in epicedrol biosynthesis’ by Madhukar S. Said *et al.*, *RSC Adv.*, 2019, **9**, 28258–28261.

We, the named authors, hereby wholly retract this *RSC Advances* article because the enzymes GPP and FPP synthase used in the paper were genetically engineered by another colleague and used without permission. In addition, the synthesis of phospholipids as described in the paper was done according to unpublished PhD thesis work and we did not have permission to publish it.

Signed: Madhukar S. Said, Govinda R. Navale, Jayant M. Gajbhiye and Sandip S. Shinde

Date: 8^th^ January 2020

Retraction endorsed by Laura Fisher, Managing Editor, *RSC Advances*

## Supplementary Material

